# Efficacy of Fascia Iliaca Compartment Blocks in Proximal Femoral Fractures in the Prehospital Setting: A Systematic Review and Meta-Analysis

**DOI:** 10.1017/S1049023X23000298

**Published:** 2023-04

**Authors:** Sabrina Slade, Evan Hanna, Josh Pohlkamp-Hartt, David W. Savage, Robert Ohle

**Affiliations:** 1. Northern Ontario School of Medicine University, Thunder Bay, Ontario, Canada; 2. Ornge Air Ambulance, Toronto, Ontario, Canada; 3. Boston Bruins, Boston, Massachusetts USA; 4. Northern Ontario School of Medicine University, Sudbury, Ontario, Canada

**Keywords:** hip fracture, peripheral nerve block, prehospital, proximal femur fracture, trauma

## Abstract

**Introduction:**

Proximal femoral fractures are characterized as one of the most common and most painful injuries sustained by patients of all ages and are associated with high rates of oligoanalgesia in the prehospital setting. Current treatments include oral and parenteral opiates and sedative agents, however regional anesthesia techniques for pain relief may provide superior analgesia with lower risk of side effects during patient transportation. The fascia iliaca compartment block (FICB) is an inexpensive treatment which is performed with minimal additional equipment, ultimately making it suitable in prehospital settings.

**Problem:**

In adult patients sustaining proximal femoral fractures in the prehospital setting, what is the effect of the FICB on non-verbal pain scores (NVPS), patient satisfaction, success rate, and adverse events compared to traditional analgesic techniques?

**Methods:**

A librarian-assisted literature search was conducted of the Cochrane Database, Ovid MEDLINE, PubMed, Ovid EMBASE, Scopus, and Web of Science indexes. Additionally, reference lists for potential review articles from the *British Journal of Anesthesia*, the *College of Anesthetists of Ireland*, the *Journal of Prehospital Emergency Care*, *Annales Francaises d’Anesthesie et Réanimation*, and the *Scandinavian Journal of Trauma, Resuscitation, and Emergency Medicine* were reviewed. Databases and journals were searched during the period from January 1, 1980 through July 1, 2022. Each study was scrutinized for quality and validity and was assigned a level of evidence as per Oxford Center for Evidence-Based Medicine guidelines.

**Results:**

Five studies involving 340 patients were included (ie, two randomized control trials [RCTs], two observational studies, and one prospective observational study). Pain scores decreased after prehospital FICB across all included studies by a mean of 6.65 points (5.25 - 7.5) on the NVPS. Out of the total 257 FICBs conducted, there was a success rate of 230 (89.3%). Of these, only two serious adverse events were recorded, both of which related to local analgesia toxicity. Neither resulted in long-term sequelae and only one required treatment.

**Conclusion:**

Use of FICBs results in a significant decrease in NVPS in the prehospital setting, and they are ultimately suitable as regional analgesic techniques for proximal femur fractures. It carries a low risk of adverse events and may be performed by health care practitioners of various backgrounds with suitable training. The results suggest that FICBs are more effective for pain management than parenteral or oral opiates and sedative agents alone and can be used as an appropriate adjunct to pain management.

## Introduction

For patients experiencing trauma, the priority is timely transportation to the most appropriate care. In Canada, nearly 22% of the population (ie, those living in rural areas) have to travel more than one hour to access a Level I or Level II trauma center.^1^ Given the distances of transport and potential for external delaying factors, travel times can often be prolonged. This requires transporting paramedics to manage the patients’ care for significant periods of time.^2,3^ Prehospital pain management is therefore a critical pillar in trauma care, and based on current available research, there are not only gaps in appropriate analgesia of the injured patient, but additional issues regarding short- and long-term sequelae secondary to oligoanalgesia.^4,5^

Proximal femoral fractures are characterized to be amongst some of the most common and most painful injuries sustained by patients of all ages and are associated with high morbidity and mortality, particularly in elderly patients.^6^ The current treatments for pain following these injuries includes oral and parenteral opiates and sedative agents that have a high risk of side effects, including: nausea, emesis, and less common but more life-threatening effects including hypotension, respiratory depression, and hypoxemia.^7^

A potential alternative to opiate medications is the use of regional anesthetic techniques conducted in the prehospital setting. Although there is a significant body of literature regarding the effectiveness of regional anesthesia in the emergency department (ED) and surgical setting, it is not commonly performed in transport or prehospital medicine.^8,9^ Regional anesthetic techniques have been proven to provide superior pain relief to oral or parenteral analgesia in the hospital setting and can reduce the overall use of opiates in patients sustaining femoral injuries.^10,11^ Additionally, new literature supports the efficacy as well as safety of peripheral nerve blocks in lower extremity trauma, including a recent retrospective cohort study including 91,563 hip fracture patients from 2009 through 2017 that concluded there was no increased risk of adverse events in patients who have received a regional block.^12^

**Figure 1.**
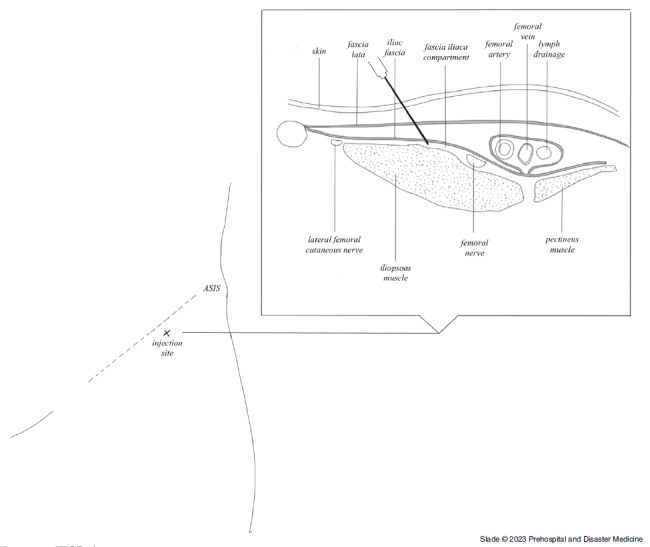
FICB Anatomy. Note: Illustration by Nusha Ramsoondar. Abbreviation: FICB, fascia iliaca compartment block.

The fascia iliaca compartment block (FICB) was first described by Dalens, et al in 1989 as a low-risk intervention that provided adequate pain relief and did not require surgical training to perform. During this study, it was also reported that this technique had superior success rates in blocking the femoral, lateral cutaneous, and obturator nerves when compared to the femoral nerve or “three-in-one block” (Figure 1). Given these characteristics, the FICB has the potential to be an effective intervention to provide appropriate analgesia in the prehospital setting.^13^

This systematic review was conducted to collect and examine current evidence available in the use of prehospital FICBs for pain control in proximal femoral injuries in the adult population; in particular, to assess if this intervention provides adequate analgesia for this setting and population and if it could be potentially implemented in routine prehospital care. The overall reduction in pain score, patient satisfaction, adverse events, and success rate were investigated using the available literature.

## Methods

### Search Strategy

**Figure 2.**
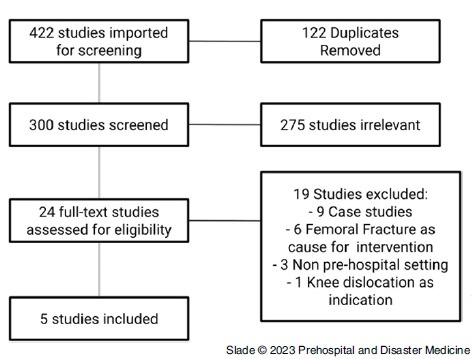
PRISMA Flow Chart.

The results are reported using the Preferred Reporting Items for Systematic Reviews and Meta-Analyses (PRISMA) guidelines^14^ (Figure 2; refer to Appendix [available online only] for search strategies).^15^

The following databases were searched: the Cochrane Database (Wiley; Hoboken, New Jersey USA); Ovid MEDLINE (US National Library of Medicine, National Institutes of Health; Bethesda, Maryland USA); PubMed (National Center for Biotechnology Information, National Institutes of Health; Bethesda, Maryland USA); Ovid EMBASE (Elsevier; Amsterdam, Netherlands); Scopus (Elsevier; Amsterdam, Netherlands); and Web of Science (Clarivate Analytics; London, United Kingdom) indexes. Additionally, reference lists for potential review articles from the *British Journal of Anesthesia*, the *College of Anesthetists of Ireland*, the *Journal of Prehospital Emergency Care*, *Annales Francaises d’Anesthesie et Réanimation*, and the *Scandinavian Journal of Trauma, Resuscitation, and Emergency Medicine* were reviewed. Language was restricted to English and French.

### Inclusion and Exclusion Criteria

*Population*—Patients ≥18 years with a Glasgow Coma Scale/GCS ≥14 with pain secondary to femoral fractures in the prehospital setting.

*Intervention*—Fascia iliaca compartment block performed in the prehospital setting by any practitioner using a technique based on landmarking.

*Comparator*—Individuals with femoral fractures who receive other forms of analgesia, including alternate regional block techniques and/or parental/oral analgesia.

*Outcomes*—The primary outcome will be pain scores pre- and post-intervention. The secondary outcomes will include complications, success rate, and patient satisfaction with the procedure.

*Study Designs*—All observational studies which use FICB in the prehospital setting and demonstrate pre- and post-analgesic data or randomized controlled trial (RCT) with two or more arms will be included.

### Study Selection

Eligibility assessments by two trained reviewers (SS, EH) were conducted independently using the systematic review software COVIDENCE (Veritas Health Innovation LTD; Melbourne, VIC, Australia). Screening was completed independently by two reviewers, and blinding was maintained throughout. Conflicts were resolved by discussion between reviewers until consensus was reached.

### Data Extraction

Once studies were screened and potential eligible studies were identified, full-text reviews were conducted. Data collection was conducted by reviewers SS and EH with information collected in a spreadsheet-based extraction table derived from the Cochrane Consumers and Communication Data Extraction Template.^16^

### Assessment of Risk of Bias and Certainty of Evidence

Each study was evaluated for its quality and validity assigning a level of evidence according to the Oxford Center for Evidence Based Medicine.^17^ Two reviewers completed risk of bias assessments without blinding for each study using the Cochrane Risk of Bias Tool for Randomized Control Trials (ROB-2) for RCTs and the Risk of Bias in Non-Randomized Studies of Interventions (ROBINS-I).^18,19^ Evidence certainty and strength was evaluated through the Grading of Recommendations Assessment, Development, and Evaluation (GRADE) approach by one reviewer (SS) and verified by a second reviewer (EH).^20^

### Data Synthesis and Statistical Analysis

To better demonstrate the differences pre- and post-intervention, or between the intervention and the comparator, mean and standard deviation (SD) in change in pain scores were extracted. Where these were not reported, they were calculated from the median, interquartile range, and minimum/maximum values.

This was conducted by using several published formulas; the limitations of these formulas as per their mathematical characteristics is that once the sample size exceeds 25, the median itself becomes the best estimate of the mean. The resultant of this was the use of the median to calculate standard deviation in replacement of a calculated mean, in accordance with the research by Hozo SP, et al.^21^ Calculations of mean and standard deviation were conducted using Microsoft Excel for Microsoft 365 MSO, Version 2201 (Microsoft Corporation; Redmond, Washington USA).

Data were synthesized using Review Manager (RevMan, Version 5.4.1; The Cochrane Collaboration; London, United Kingdom). Individual and pooled odds ratios (ORs) with 95% confidence intervals (CIs) were calculated for each of the outcomes of interest using all studies with sufficient data. This was conducted using a random effects meta-analysis due to the expected significant heterogeneity between studies. Heterogeneity was assessed through the I^2^ statistic using criteria from the GRADE handbook: low, ≤40%; moderate, 30%-60%; substantial, 50%-90%; or considerable inconsistency, ≥75%.^17^ Publication bias was assessed through a funnel plot.

## Results

This search yielded 300 titles and abstracts for review. A total of five studies (two RCTs and three prospective observational studies) met the inclusion criteria (Table 1).

**Table 1.** Included Studies

Author (year)	Study Design	Intervention	Median Age	N Subjects (Sex - % Female)	Change NVPS	Adverse Events	Patient Satisfaction	Success Rate
Jones, et al (2019)	RCT	FICB	81	31 (80.60%)	n/a*	1	3.4/4	n/a
		SOC	82	26 (76.90)	n/a*	4	3.5/4	n/a
McRae, et al (2015)	RCT	FICB	81	11 (6%)	7.5	0	n/a	81%
		SOC	83	13(10%)	5.7	7	n/a	–
Dochez, et al (2014)	POCT	FICB	81	100 (80%)	5.3	7	9/10	96%
Gros, et al (2014)	POCT	FICB	29	63(31%)	6.8	0	n/a	87%
Gozlan, et al (2005)	POCT	FICB	64	52(62.5%)	7	1	n/a	94%

Note: Please refer to appendix (available online only) for unabridged version.

Abbreviations: NVPS, non-verbal pain score; RCT, randomized controlled trial; POCT; prospective observational study; FICB, fascia iliaca compartment block; SOC, standard of care.

Pain scores decreased after prehospital FICB across all included studies, with several of the studies demonstrating statistically significant changes. Out of the total 257 FICBs conducted, only two serious adverse events were recorded, both of which related to local analgesia toxicity. Neither resulted in long-term sequelae and only one required treatment (Table 2).

**Table 2.** Quality Assessment via Cochrane Risk of Bias Tool for Randomized Control Trials (ROB-2)

Author	Sequence Generation	Allocation Concealment	Blinding – Participants/Personnel	Blinding- Outcome Assessment	Incomplete Outcome Data	Selective Reporting	Other Sources of Bias	Overall Comments
Jones, et al	Low	Low	High	Low	Low	Low	N/A	Allocation done by scratch cards provided to participants. Given the nature of the intervention, it is not possible to blind patients/participants once allocated to the trial arm. However, the second pain score was conducted by an ER triage nurse who was blinded to a trial arm.
McRae, et al	High	Unsure	High	High	Low	Low	N/A	Unclear method of allocation to trial arms. Testing of blocks conducted by individuals who conducted blocks.

Abbreviation: ER, emergency room.

### Primary Outcome

**Figure 3.**
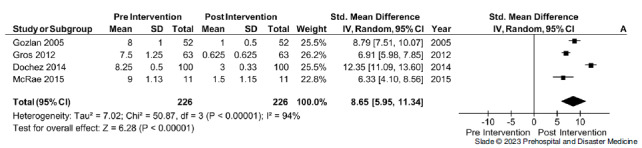
Change in Non-Verbal Pain Scale Pre- and Post-Intervention.

**Figure 4.**
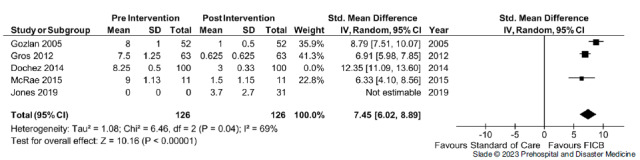
Sensitivity Analysis. Abbreviation: FICB, fascia iliaca compartment block.

*Effect on Pain Score—*Four studies provided data on a change in pain score; the non-verbal pain score (NVPS) following FICB had a statistically significant decrease in pain scores following the intervention.^22^ Within the included studies, a total of 226 FICBs were conducted, with a mean improvement of six points and a standard mean difference of 8.65 (95% CI, 5.95 to 11.34; Figure 3). This affect was noted to be consistent across observational and RCTs used within the study. Overall, heterogeneity was high (I^2^ = 94%); sensitivity analysis demonstrated that heterogeneity was accounted for by one trial. Removal of the study by Dochez, et al had no effects on the summary estimate, but heterogeneity was reduced to 69% (Figure 4, Table 3).

**Table 3.** Quality Assessment via Risk of Bias in Non-Randomized Studies of Interventions (ROBINS-I) Tool for Observational Studies

Author	Risk of Bias due to Confounding	Risk of Bias in Selection of Participants	Risk of Bias in Classification of Interventions	Bias due to Deviations from Intended Interventions	Bias due to Missing Data	Bias in Measurement of Outcomes	Bias in Selection of the Reported Result	Overall Risk of Bias	Comments
Dochez, et al	Low	Low	Low	Low	High	High	Low	Low	Block effectiveness was tested by the same individual which conducted the FICB, resulting in a high level of potential outcome assessment bias.
Gros, et al	High	Low	High	High	Low	High	Low	High	There was no specific allocation to any arm. Physicians were able to select the block technique, and anesthetic medications as they wished. Additionally, the physicians tested their own blocks which can contribute to the outcome assessment bias.
Gozlan, et al	High	Low	Low	Low	High	Low	Low	Low	Given the type of study there was no comparator and thus patients were not allocated. Treatment was also not concealed from the patient. However, a separate individual conducted testing of the block.

Abbreviation: FICB, fascia iliaca compartment block.

**Figure 5.**
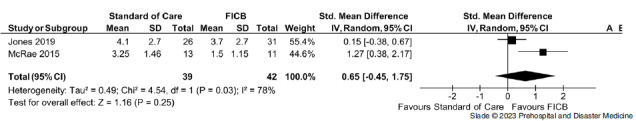
Pain Control Standard of Care vs FICB. Abbreviation: FICB, fascia iliaca compartment block.

Differences in post-intervention pain scores between arms of the RCTs conducted by Jones, et al and McRae, et al favored the use of the FICB. However, these results demonstrated a low overall effect (P = .03) and a standard mean difference of 0.65 (95% CI, -0.45 to 1.75; Figure 5).

### Secondary Outcomes

*Success Rate—*The definition of success in providing regional anesthesia in the studies examined varied greatly. All studies used hot/cold proprioception in the anterior, medial, and lateral thigh to determine success of the intervention. The study by Dochez, et al^23^ defined a block to be successful if there was an overall decrease in the pain score by at least four points on the NVPS. They also reported 88 patients (88%) had a loss of sensation within the femoral nerve distribution. Alternatively, McRae, et al^11^ defined success if two or more nerve distributions had changes in sensation. Whereas Gros, et al^24^ defined blocks to be successful (ie, complete or incomplete) if there were any changes in the patient’s sensation. The study by Gozlan, et al^25^ defined success if the block covered all three nerve distributions. Across these studies, the success rate was 89.3% or 230 successful blocks out of 257 attempts. The study conducted by Jones, et al^26^ did not disclose their success rate, and as result, was included in the analysis as an unsuccessful attempt given the lack of data (Table 1).

*Adverse Events—*Within the five included studies, a total of 257 FICBs were performed in the prehospital setting by a variety of health care professionals. This included 42 by paramedics, 100 by ED nurses, and 159 by physicians (ie, emergency physicians or anesthesiologists). There was a total of two major adverse events across the five studies in the experimental group, both of which were related to local anesthetic toxicity. The first severe adverse event was noted in the study by Gozlan, et al^25^ where the patient experienced a transient episode of tachycardia and hypertension lasting approximately ten minutes after receiving the block; however, these symptoms resolved spontaneously within five minutes. The other case detailed by Jones, et al^26^ required the use of Intralipid. In both cases, neither patient experienced any long-term sequelae. Of note, seven minor adverse events were observed relating to nausea and occurred following the use of parenteral opiates (Table 1).

*Patient Satisfaction—*Three of the five included studies discussed patient satisfaction with analgesia. The first by Dochez, et al^23^ used a regional-based pain scoring system from one (absolutely not satisfied with care) to ten (very satisfied with care). Patients reported high satisfaction from both groups with a median score of nine.

The paper by McRae, et al^11^ used a five-point scale to assess patient satisfaction with analgesia with rating options of nil, poor, average, good, and excellent. Between the FICB group and the control group, there was found to be no significant difference; one patient in the FICB group reported quality of analgesia as average, with no patients in either group rating their satisfaction as poor or nil. Unfortunately, a qualitative analysis of the breakdown of scoring was not available.

Lastly, the study by Jones, et al^26^ rated patient satisfaction with analgesia on a four-point scale, but the ratings for their scale were not shared. There was no stated clinical difference between the control group and the experimental group with mean satisfaction scores of 3.5 and 3.4 out of a possible 4.0, respectively (Table 1).

## Discussion

### Interpretation

The studies examined demonstrated that overall pain control is significantly improved and favors the FICB intervention. The Tau^2^ and Chi^2^ analysis with 95% confidence intervals provided significant data heterogeneity amongst the studies included. This level of heterogeneity creates a challenge for overall interpretation of the meta-analysis. An interesting note from the Gros, et al 2012 study was that the standard mean difference was significantly higher than the others; it is hypothesized that this is secondary to training protocols for the FICBs. The training method of the ED nurses who conducted the regional anesthesia appears to be more rigorous than the other studies, and as a result, contributed to a higher standard mean difference.

Based on the results of Figure 5, which includes studies by Jones, et al and McRae, et al which are the only RCTs in this meta-analysis, the evidence does suggest an overall effect, but clinically, the standard mean difference between the treatment methods would not be considered statistically significant.^22^ Both studies demonstrate high heterogeneity which provides minimal evidence for the benefit of FICB compared to the current standard of care; however, this effect can be explained by the low number of participants included in the trials. With an increased number of participants, there would be an anticipated improvement in heterogeneity and effect. Inclusion of the observational studies results in favoring the FICB compared to standard of care; however, it is difficult to come to a firm conclusion given the lower quality observational studies and overall high heterogeneity.

There is a lack of qualitative data examining the effects of FICB on patient satisfaction, but the general quantitative findings from the studies examined is that it appears to be relatively similar between control and experimental groups. Additionally, only one study assessed long-term outcomes of patients receiving FICB.^26^

Generally, high success rates of achieving anesthesia from the nerve block were seen across all studies; this is a strong reflection of the simplicity of the block and its suitability for low-resource settings and use by practitioners of various training backgrounds. It is important to note that there was a lack of consistency between the studies for determining the overall success of the block, and should be interpreted cautiously and in a subjective manner to take into account pain score as well as patient satisfaction.

Although documented complications in the FICB group were quite low, there were still two incidences of local anesthetic toxicity. Anesthetic toxicity is one of many potential risks of regional anesthesia; other complications include nerve injury (ie, temporary or permanent), infection, allergic reaction, and bleeding. Not surprisingly, a significantly higher number of adverse events were noted within the standard of care arm in the RCTs, most of which were characterized as respiratory depression secondary to opiate use.^26^

## Limitations

The major limitation to this review is the number of studies; given the relatively small sample size, only extrapolation of the potential outcomes is achievable and adverse events secondary to the intervention are unable to be detected. In addition to there being a limited number of studies, three of them were observational. These studies are at risk of confounding (specifically residual confounding) and selection bias; although they were deemed low risk using the ROBINS-I tool, the results could have potentially been skewed to favor the desired outcome. An attempt was made to assess publication bias, however due to the small number of studies, a funnel plot could not be constructed. Another important issue is the use of concurrent analgesia. All studies provided patients with adjunct oral or parenteral pain control in addition to the block; this could potentially limit the patient’s ability to accurately assess pain control. Lastly, although the techniques were the same, the quantity and type of local anesthetics were different, which can pose issues when comparing NVPS ratings among studies.

## Conclusions

The use of FICBs results in a significant decrease in NVPS in the prehospital setting and are ultimately suitable as a regional analgesic technique for proximal femur fractures. It carries a low risk of adverse events and may be performed by health care practitioners of various backgrounds with suitable training. Results suggest that FICBs are more effective for pain management than parenteral or oral opiates and sedative agents alone, and can be used as an appropriate adjunct to pain management. Further research is, however, required to provide additional quantitative evidence in the use of FICBs independent of current standard of care practices.

## Supplementary material

For supplementary material accompanying this paper visit https://doi.org/10.1017/S1049023X23000298. click here to view supplementary material
