# Supplementary material for: Efficacy of Fascia Iliaca Compartment Blocks in Proximal Femoral Fractures in the Prehospital Setting: A Systematic Review and Meta-Analysis
Source: Prehosp Disaster Med. 2023 Mar 13;38(2):252–8. doi: 10.1017/S1049023X23000298 (PMC10027490; doi:10.1017/S1049023X23000298)
Supplement: Supplementary file 1 [file S1049023X23000298sup001.docx]

Appendix Table 1a.

Quality Assessment via Cochrane Risk of Bias Tool for Randomized Control Trials

| Author | Sequence Generation | Allocation Concealment | Blinding – Participants/ Personnel | Blinding- Outcome Assessment | Incomplete Outcome Data | Selective Reporting | Other sources of Bias | Overall Comments |
| --- | --- | --- | --- | --- | --- | --- | --- | --- |
| Jones et al. | Low | Low | High | Low | Low | Low | N/A | Allocation done by scratch cards provided to participants. Given the nature of the intervention, it is not possible to blind patients/ participants once allocated to the trial arm. However, the second pain score was conducted by an ER triage nurse who was blinded to a trial arm. |
| McRae et al | High | Unsure | High | High | Low | Low | N/A | Unclear method of allocation to trial arms. Testing of blocks conducted by individuals who conducted blocks. |

Table 1b

Quality assessment via ROBINS-I tool for observational studies

| Author | Risk of Bias due to confounding | Risk of Bias in selection of participants | Risk of Bias in classification of interventions | Bias due to deviations from intended interventions | Bias due to missing data | Bias in measurement of outcomes | Bias in selection of the reported result | Overall risk of Bias | Comments |
| --- | --- | --- | --- | --- | --- | --- | --- | --- | --- |
| Dochez et al | Low | Low | Low | Low | High | High | Low | Low | Block effectiveness was tested by the same individual which conducted the FICB, resulting in a high level of potential outcome assessment bias. |
| Gros et al | High | Low | High | High | Low | High | Low | High | There was no specific allocation to any arm. Physicians were able to select the block technique, and anesthetic medications as they wished. Additionally, the physicians tested their own blocks which can contribute to the outcome assessment bias. |
| Gozlan et al | High | Low | Low | Low | High | Low | Low | Low | Given the type of study there was no comparator and thus patients were not allocated. Treatment was also not concealed from the patient. However, a separate individual conducted testing of the block. |

Appendix Table 2. Characteristics of Included Studies

| Author, Year | Study Design | Population | N Subjects | % Female | Median Age | Intervention | Comparator | Primary Outcome:  Pain Control (out of 10 based on Verbal Numerical Pain Rating Score) | Secondary Outcomes: | | |
| --- | --- | --- | --- | --- | --- | --- | --- | --- | --- | --- | --- |
|  |  |  |  |  |  |  |  |  | Adverse Events | Patient Satisfaction | Success Rate of FICB |
| Jones et al.  2019 | Randomized Control Trial | Adults ≥ 18 years with high clinical suspicion of hip fracture | FICB: 31  SOC :26 | FICB: 80.60%  SOC: 76.90% | FICB 81.5  SOC: 82.2  *provided as mean | Blind FICB “2 pop technique” using 20ml of 1% prilocaine | UK Standard of care hip fracture protocol: weight based  Paracetamol and morphine | Post intervention:  FICB: 3.7 (SD 2.7)  SOC: 4.1 (SD 2.7) | FICB: 1 patient experienced Local anesthetic toxicity required treatment with intralipid. 2 died within 7 days due to unrelated causes.  SOC: 1 patient experienced rhabdomyolysis, 2 died within 7 days of injury due to heart failure, and 1 required multiple blood transfusions | Both groups reported satisfactory pain control.  Mean satisfaction of 4 point scale:  FICB: 3.4/4  SOC: 3.5/4 | Not observed |
| McRae et al.  2015 | Randomized Control Trial | Adults ≥ 18 years with high clinical suspicion of femoral fracture (proximal or shaft) | FICB: 11  SOC: 13 | FICB: 6%  SOC: 10% | FICB: 81 (70-85)  SOC 83 (73-88) | Blind FICB “2 pop technique” using 30-40ml of a solution composed of 20ml 2% lidocaine + 20ml epinephrine diluted 1:200,000 | Weight based IV Morphine Sulfate (2.5mg q2min) to a maximum dose of 0.5mg/kg | Pre Intervention  FICB:  9 (SD 1.13)  SOC:  9 (0.61 SD)  Post Intervention:  FICB 1.5 (SD 1.15)  SOC: 3.25 (SD 1.46) | FICB: No adverse events  SOC:  38% of patients experienced nausea  2 episodes of respiratory depression (attributed to opiate use) | Assessed on a 5 point scale, both arms reported high satisfaction. Qualitative results were unavailable. | Defined success as 2 or more distributions with changes in sensation    81% success:    45% full block    36 % partial block |
| Dochez et al.  2014 | Prospective Observational | Adults ≥ 18 years with high clinical suspicion of femoral fracture ( proximal or shaft) | FICB : 100 | 80% | 81 (SD 9.4)  *provided as Mean | Blind FICB “2 pop technique” utilizing dosing 0.3ml/kg 1% lidocaine with 5ug/ml epinephrine | None | Pre Intervention:  8.25 (SD 0.5)  Post intervention:  3 (SD 0.33) | 7 patients reported nausea during transportation | Median score of 9/10 for patient satisfaction | Defined as success if decrease in pain score by at least 4 points.  96% success rate, 88% of patients had decreased sensation in femoral nerve distribution. |
| Gros et al.  2012 | Multi-Arm Prospective Observational | Any patient with likely hip or femur fracture | 63 FICB  8 FNB  36 FBNS | 31% | 29 (14-70) | Blind “2 pop technique” FICB. (FICB)  Discretion to amount and type of local anesthetic left to practitioner | Blind Femoral Block “2 pop technique” (FNB), Femoral block with nerve stimulator (FBNS)  Discretion to amount and type of local anesthetic left to practitioner | Pre Intervention:  FICB: 7.5 (SD 1.25)  FNB: 8.1 (SD 0.72)  FBNS: 7.5 (SD 1.25)  Post Intervention:  FICB 0.625 (SD 0.625)  FNB: 1.25 (SD 0.75)  FBNS: 0.625 (SD 0.625) | None | Not discussed | Defined as success if complete block present in all nerve distributions.  FICB : 55/63 successful  8/63 failed, however 2 additional blocks were successful following the second attempt.  FNB: 8/8 success  FBNS: 34/36 success |
| Gozlan et al  2005. | Prospective Observational Study | Any patient with likely hip of Femur Fracture | FICB: 52 | 62.5% | 64 (SD 28)  *provided as mean | FICB performed with “2 pop technique” using 0.4ml/kg of 1.5% lidocaine with epinephrine 1:400,000. (Mean dose of 23ml) | None | Pre Intervention:  8 (SD 1)  Post Intervention:  1 (SD 0.5) | Local anesthetic reaction in one patient: Transient tachycardia and hypertension. Resolved spontaneously 10 mins post intervention without treatment. | Not discussed | Successful if complete block.  49/52 complete sensory block  2/52 incomplete sensory block 2/3 territories  1/52 incomplete sensory block 1/3 territories |
